# Molecular and Metabolic Mechanisms Underlying Selective 5-Aminolevulinic Acid-Induced Fluorescence in Gliomas

**DOI:** 10.3390/cancers13030580

**Published:** 2021-02-02

**Authors:** Jeffrey I. Traylor, Mark N. Pernik, Alex C. Sternisha, Samuel K. McBrayer, Kalil G. Abdullah

**Affiliations:** 1 Department of Neurological Surgery, University of Texas Southwestern Medical Center, Dallas, TX 75235, USA; jeffrey.traylor@utsouthwestern.edu (J.I.T.); mark.pernik@utsouthwestern.edu (M.N.P.); 2 Children’s Medical Center Research Institute, University of Texas Southwestern Medical Center, Dallas, TX 75390, USA; alex.sternisha@utsouthwestern.edu

**Keywords:** 5-ALA, high-grade glioma, low-grade glioma, intraoperative fluorescence, protoporphyrin IX

## Abstract

**Simple Summary:**

5-aminolevulinic acid (5-ALA) is a medication that produces fluorescence in certain cancers, which enables surgeons to visualize tumor margins during surgery. Gliomas are brain tumors that can be difficult to fully resect due to their infiltrative nature. In this review we explored what is known about the mechanism of 5-ALA, recent discoveries that increase our understanding of that mechanism, and potential targets to increase fluorescence in lower grade gliomas.

**Abstract:**

5-aminolevulinic acid (5-ALA) is a porphyrin precursor in the heme synthesis pathway. When supplied exogenously, certain cancers consume 5-ALA and convert it to the fluorogenic metabolite protoporphyrin IX (PpIX), causing tumor-specific tissue fluorescence. Preoperative administration of 5-ALA is used to aid neurosurgical resection of high-grade gliomas such as glioblastoma, allowing for increased extent of resection and progression free survival for these patients. A subset of gliomas, especially low-grade tumors, do not accumulate PpIX intracellularly or readily fluoresce upon 5-ALA administration, making gross total resection difficult to achieve in diffuse lesions. We review existing literature on 5-ALA metabolism and PpIX accumulation to explore potential mechanisms of 5-ALA-induced glioma tissue fluorescence. Targeting the heme synthesis pathway and understanding its dysregulation in malignant tissues could aid the development of adjunct therapies to increase intraoperative fluorescence after 5-ALA treatment.

## 1. Introduction

Glioma is the most common primary malignancy of the central nervous system and is classified as grades I–IV by the World Health Organization (WHO). In general, grade I and II gliomas are considered “low-grade” (LGG) and comprise astrocytoma (pilocytic and diffuse subtypes) and oligodendrogliomas, while grade III and IV lesions, including anaplastic astrocytoma and glioblastoma (GBM), are considered “high-grade” (HGG) [1]. Generally, LGG is less aggressive and confers a better overall prognosis. However, virtually all LGG tumors progress to HGG and eventually GBM which confers a particularly poor outcome. Despite resection with adjuvant chemoradiation having been shown to improve survival in patients with GBM, median survival is still approximately 14 months from the time of diagnosis [2]. Since care was standardized to adjuvant temozolomide and radiation 15 years ago, little progress has been made in improving the prognosis of glioma patients. However, several prognostic factors have been identified that have led to more accurate risk stratification. The relationship between extent of resection (EOR) and survival is one such prognostic factor that is becoming increasingly evident. Specifically, maximum resection of the contrast-enhancing portion of high-grade tumors confers longer overall survival [3,4,5,6,7]. Moreover, there is emerging evidence to suggest that even supra-total resection of regions beyond the contrast-enhancing margin may confer a survival benefit in GBM, further underscoring the importance of EOR in HGG [8]. The same relationship has been elucidated for LGG, although this is defined by mostly retrospective evidence [9,10].

A hallmark of low- and high-grade glioma alike is the propensity to infiltrate into surrounding tissue [11]. Unlike central nervous system (CNS) metastases which are generally well circumscribed and exert a mass effect on adjacent neurovascular structures, glioma invades and disrupts this anatomy, making en bloc gross total resection challenging. In HGG cases, attempts are made to resect the contrast-enhancing margin corresponding to contrast-enhancement on magnetic resonance imaging (MRI). Stereotactic navigation can be used to ensure that the resection margin extends to this contrast-enhancing border on preoperative imaging.

As visualizing the tumor margins for infiltrating regions of glioma in the operating room is difficult, techniques for intraoperative fluorescence have emerged to improve EOR in this patient population. 5-aminolevulinic acid (5-ALA) is the most prominent and well-studied of these and has been shown to increase EOR and progression-free survival in malignant glioma based on the results of a 2006 phase III trial [5]. The authors of this trial ultimately concluded that 5-ALA enabled more complete resection of the contrast-enhancing tumor. Of note, tumors with substantial non-contrast-enhancing regions, consistent with LGG, were excluded from analysis due to poor fluorescent response to 5-ALA administration. In fact, only 10–20% of LGG exhibited visible fluorescence with 5-ALA, making EOR in this patient population challenging [12,13]. Additionally, the use of 5-ALA has been associated with sporadic but not consistent fluorescence in some oncologic subtypes during resection, including metastatic lesions and lymphoma [14,15,16,17]. This indicates that in contrast to both LGG and other cranial tumors, the uniform and robust fluorescence found in high-grade gliomas represents a distinct and specific biological mechanism. In this systematic review, we discuss the underlying molecular mechanisms for 5-ALA fluorescence in glioma and summarize previous attempts at targeting these mechanisms with pharmacologic supplementation and photodynamic therapy. This review was conducted in accordance with the preferred reporting items for systematic reviews and meta-analyses (PRISMA) statement [18].

## 2. 5-ALA Development and History

**Figure 1:**
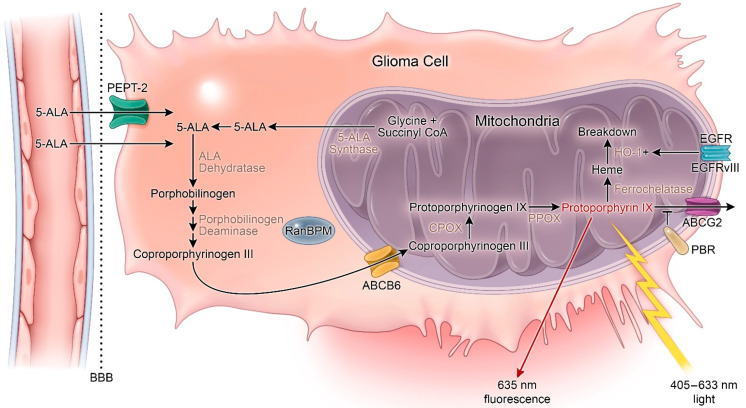
Heme synthesis pathway and mechanism of 5-aminolevulinic acid (5-ALA) fluorescence. 5-ALA: 5-aminolevulinic acid; ABCG2: ATP-binding cassette G2; ABCB6: ATP-binding cassette B6; BBB: Blood–brain barrier; CPOX: Coproporphyrinogen oxidase; HO-1: Heme-oxygenase 1; PBR: Peripheral benzodiazepine receptor; PPOX: Protoporphyrinogen oxidase. + = increased activity.

5-ALA is a non-proteinogenic amino acid formed through the condensation of succinyl-CoA and glycine that serves as a metabolic precursor for heme biosynthesis. In cells, 5-ALA is converted to protoporphyrin IX (PpIX), a photosensitizer and direct precursor to hemoglobin in the heme synthesis pathway. As a photosensitizer, PpIX is excited by light between *λ* = 405 (violet) and *λ* = 633 nm (red) [19]. The pathway for 5-ALA uptake and conversion to PpIX is outlined in Figure 1. Following initial discovery and synthesis of 5-ALA, it was tested for the diagnosis and treatment of skin, gastrointestinal, and bladder cancers, where it aided in disease recognition, excision, and photosensitizing treatment [20,21,22,23]. In 1998, Stummer and colleagues first described their results using oral 5-ALA to enhance intraoperative fluorescence of HGG which displayed high sensitivity, specificity, and accuracy for malignant cells [24]. Notably, their sentinel manuscript reported a lack of fluorescence in one patient with an LGG [24].

To build on this discovery, Stummer and colleagues went on to conduct a randomized phase III trial. They concluded that intraoperative use of 5-ALA in HGG was safe and markedly increased complete resection rates (65% in 5-ALA vs. 36% control). However, their study was underpowered to detect a difference in overall survival [5,25]. Based on these findings, the European Medicines Agency approved the use of 5-ALA in 2007. However, the United States Food and Drug Administration (FDA) approval took significantly longer due to their view of 5-ALA as a therapeutic rather than an adjunct tool to aid intraoperative visualization [26]. After the approval of an orphan drug application and the publication of other trials that highlighted the utility of 5-ALA in improving sensitivity and specificity of tumor margin discrimination during HGG resection, as well as progression-free survival at 6 months, the drug was approved for use in WHO grades III or IV by the FDA in 2017 [13,25,27,28,29].

5-ALA is the most studied of the photosensitizing agents in glioma and possesses many desirable properties for intraoperative tumor tissue discrimination. First, it is rapidly eliminated, reducing risk of skin sensitivity [30]. Indeed, early studies of 5-ALA metabolism demonstrated that approximately 80% of the substance was excreted in 24 h compared to ^15^N-labeled L-α-amino acids in rats [31]. Second, 5-ALA is readily bioavailable following treatment with oral formulations. In patients undergoing cranial surgery, patients are administered 5-ALA as a well-tolerated oral liquid 2–4 h before craniotomy. Third, 5-ALA accumulates in very few normal tissues (including mucosa of gastrointestinal tract, salivary glands, bile ducts, and seminal vesicles), thereby contributing to high signal to noise characteristics for tumor tissue visualization applications [22,32]. Fourth, 5-ALA is an endogenous human metabolite that is synthesized naturally from succinyl-CoA and glycine in the mitochondria; therefore, 5-ALA treatment is not associated with toxic side effects associated with administration of many xenobiotics. Taken together, these properties cooperate to render 5-ALA a valuable adjunct to neurosurgical HGG resection [33].

## 3. Mechanism of 5-ALA Fluorescence

In normal cells, succinyl-CoA and glycine undergo condensation by 5-ALA synthase to form 5-ALA. 5-ALA is then converted through a series of enzymatic steps to PpIX and, ultimately, to heme. This process is regulated by free iron and heme. PpIX accumulation in the cell can result from (1) increased 5-ALA levels, (2) 5-ALA synthase hyperactivity, or (3) dysfunction of the ferrochelatase (FECH) enzyme that produces heme from PpIX [34]. Impaired tumoral FECH activity is one potential explanation for the specificity of PpIX accumulation in tumor tissues following 5-ALA treatment. Indeed, FECH has been shown to be decreased in colon carcinoma liver metastases compared to liver parenchyma in rat models following 5-ALA administration. Similar results were also reported in prostate cancer, bladder cancer, and colonic cells [35,36,37].

Porphobilinogen deaminase (PBGD) is another enzyme in the heme-synthesis pathway that promotes PpIX production. Increased levels of PpIX corresponding to increased activity of PBGD as well as ALA dehydratase and uroporphyrinogen decarboxylase have been reported in human breast carcinoma cells [38]. Similar results have been reported for squamous cell carcinoma and adenocarcinoma [39]. Additional evidence indicates that the feedback mechanism between PBGD and ALA dehydratase may play a central role in PpIX synthesis [40]. In 2002, Greenbaum et al. described a significant subcellular localization of PBGD in the nucleus that rapidly decreased following stimulation of C6 glioma cell differentiation [41,42]. The authors concluded that PBGD may have a unique nuclear function in glioma cells. One year later, these investigators identified the nuclear Ran-binding protein RanBPM as an interacting partner of PBGD [43]. They further concluded that nuclear localization of PBGD in glioma may be related to the process of malignant transformation in glioma. Although these results implicate altered activity of heme biosynthesis pathway enzymes as a general cause of 5-ALA-induced PpIX accumulation in malignant tissue, the mechanistic underpinning of this phenotype specifically relevant in HGG is an area of intense investigation.

Coproporphyrinogen oxidase (CPOX) is an enzyme of the heme synthesis pathway that generates protoporphyrinogen III (a direct precursor from PpIX) from coproporphyrinogen III via oxidative decarboxylation. Expression of this enzyme has been shown to directly correlate with PpIX fluorescence in human glioma cells [44].

In the setting of GBM, FECH expression has been shown to be reduced relative to normal brain tissue, leading to accumulation of PpIX. Importantly, FECH silencing via RNA interference is sufficient to promote this phenotype, thereby providing evidence of a functional connection between FECH repression and 5-ALA-induced PpIX accumulation in GBM [45]. Similar results linking PpIX accumulation and decreased FECH activity have been shown in medulloblastoma cell lines [46]. In addition to regulation at the level of gene expression, FECH can also be repressed by iron chelation. Reduced intracellular iron content blocks FECH activity and triggers PpIX accumulation in human adenocarcinoma and hamster lung fibroblast cell lines [47,48,49,50]. Differentiation therapy targets another enzyme, coproporphyrinogen oxidase, in the heme synthesis pathway, leading to increased PpIX concentration in prostate cancer cells [51]. Unfortunately, no differentiation therapies exist for malignant gliomas as these tumors are known to have a partial and abnormal potential for differentiation [52].

## 4. Metabolic Activity

Further insight into the mechanism of 5-ALA fluorescence has been elucidated through studies of heme metabolism and its link to the tricarboxylic acid (TCA) cycle. Differential fluorescence upon 5-ALA treatment was observed in the IDH1^R132H^ mutant compared to IDH1 wild-type (WT) WHO grade III gliomas. In IDH1^R132H^ gliomas, the metabolite R-2-hydroxyglutarate (2-HG) accumulates, leading to cellular reprogramming and oncogenesis. The observation in gliomas was further explored in U87MG-IDH1^R132H^ cells in comparison to U87MG-IDH1^WT^ cells, where U87MG- IDH1^R132H^ cell metabolism of 5-ALA to PpIX was delayed in contrast to U87MG- IDH1^WT^ cells. As TCA cycle metabolites are involved in the generation of 5-ALA and thus PpIX, an exploration of the metabolic events that underlie the differences in 5-ALA mediated fluorescence was explored [53].

Upon 5-ALA treatment in U87MG-IDH1^R132H^ cells, citrate and 2-HG concentrations were significantly increased, while α-ketoglutarate (α-KG) decreased relative to U87MG- IDH1^WT^ cells. From these data, Kim et al. reviewed the interconnectedness of the TCA cycle and the heme biosynthesis pathway and hypothesized that nicotinamide adenine dinucleotide phosphate (NADPH)-dependent heme degradation is impaired, based on knowledge that the IDH1^R132H^ mutation perturbs NADPH homeostasis [53,54]. Specifically, this mutation abrogates WT activity of the IDH1 enzyme, thereby blocking NADPH synthesis associated with the oxidative decarboxylation of isocitrate to α-KG. Furthermore, the IDH1^R132H^ oncoprotein consumes NAPDH in the process of producing 2-HG [55]. Reduced levels of NADPH in IDH-mutated glioma cells has been proposed to contribute to PpIX accumulation [53]. Although this study highlighted metabolic alterations that lead to variance in 5-ALA-mediated fluorescence in IDH-mutated glioma cells, it is still not clear how IDH mutations influence glioma tissue fluorescence following 5-ALA treatment in the clinical setting. In particular, the simultaneous high prevalence of IDH mutations and low 5-ALA-induced tissue fluorescence associated with LGGs appear to be in conflict with the thesis that mutant IDH action stimulates PpIX content in glioma.

This conflict notwithstanding, there is additional evidence that NADPH production capacity may influence 5-ALA fluorescence due to crosstalk between the TCA cycle and heme synthesis pathway. Kim et al. conducted an RNA-sequencing study to compare GBM types classified by 5-ALA fluorescence [56]. This group identified glutaminase 2 as a regulator of 5-ALA fluorescence and confirmed this role in functional in vitro assays. Reduced expression of glutaminase 2 was associated with decreased production of NADPH and increased levels of PpIX and reactive oxygen species (ROS) in regions of GBM tumors with high fluorescence [56].

Lastly, lentiviral shRNA-mediated silencing of the heme biosynthesis enzymes in breast cancer cells was explored to better understand the pathway’s role in 5-ALA fluorescence. Porphobilinogen synthase (PBGS) and porphobilinogen deaminase are involved in the production of PpIX, whereas FECH degrades PpIX. Silencing of PBGS and PBGD led to decreased 5-ALA-mediated fluorescence and decreased sensitivity to photodynamic therapy (PDT), whereas silencing of FECH led to the opposite effects on fluorescence and PDT sensitivity [57].

### 4.1. Membrane Transport

Uptake of exogeneous 5-ALA via active transport is cell-specific and provides negative feedback to 5-ALA synthase and accumulation of PpIX in the cell. PpIX efflux from the cell has also been studied and shown to be dependent on several factors. PpIX is synthesized in the mitochondria but quickly transports to the cytosol [58]. The mitochondrial peripheral benzodiazepine receptor (PBR) is one possible mechanism that has been shown to contribute to PpIX accumulation. In one study, PpIX concentration diminished with the administration of a competitive peripheral benzodiazepine receptor blockade concurrently with 5-ALA [59]. Another study showed that induction of PBR in glioma cell lines caused corresponding increases in intracellular PpIX concentration [60]. ATP-binding cassette transporter G2 (ABCG2) is another transporter that has been studied in the context of PpIX, with an inverse correlation between transporter expression and 5-ALA accumulation, although ABCG2 may be differentially expressed in GBM [58,61,62]. Further, cadherin 13 has been reported as a potential regulator of ABCG2 and PEPT1 expression and, accordingly, PpIX accumulation in glioma cells [45]. Overexpression of another ATP-binding cassette transporter known to regulate porphyrin synthesis, ABCB6, has also been shown to increase PpIX accumulation in glioma cell lines and subsequently increase PDT effects [63]. Epidermal growth factor receptor (EGFR) and EGFRvIII co-expression has been shown to influence 5-ALA fluorescence in GBM [64]. Additionally, PpIX efflux out of the cell was reported in the presence of fetal bovine serum [65].

It is estimated that up to 20% of LGG will have macroscopic fluorescence after 5-ALA administration [12]. As pointed out by Collaud et al., the slowest step between 5-ALA uptake and formation of PpIX must be faster than the conversion of PpIX to heme in order for PpIX to accumulate and significant fluorescence to occur [66]. The variables that lead to 5-ALA-induced PpIX accumulation in target tissue are numerous and include blood–brain barrier (BBB) integrity, expression of membrane transporters and heme biosynthesis pathway enzymes, as well as tissue environmental factors (temperature, pH, etc.) which are reviewed in Table 1.

### 4.2. Blood–Brain Barrier

Gadolinium contrast-enhancement in malignant glioma is reliant on disruption of the BBB and is typically not reflected by infiltrating regions of the tumor margin [82]. However, PpIX has been shown to accumulate following 5-ALA administration in infiltrating glioma cells [68] and has been shown to be more sensitive for detecting active tumors than contrast or molecular imaging [68,83]. 5-ALA uptake in the CNS is believed to occur via proton-dependent peptide transporter 2 (PEPT2) and a putative Na^+^/HCO_3_^−^ organic anion transporter in the choroid plexus [67]. PEPT2 helps transport oligopeptides and peptide-like molecules throughout the body and is an important driver of 5-ALA accumulation in non-glioma cancers alike [84,85]. Specifically, the Na^+^/HCO_3_^−^ transporter may work to impact cellular pH, thus decreasing 5-ALA accumulation via the proton-dependent PEPT2 channel [67]. PEPT2 levels tend to be increased in HGG compared to LGG [86]. Consistently, in an analysis of grade II and III gliomas, expression of mRNA encoding for PEPT2 was significantly higher in fluorescent tumors compared to non-fluorescent [87].

There is some evidence to suggest that the BBB plays a role in PpIX tumor selectivity in malignant glioma, specifically [33]. Several studies have shown that the intact BBB is relatively impermeable to 5-ALA, although liposomal encapsulation has been shown to facilitate transport and increase porphyrin concentration in the brain [70,71,72,75]. Further, it is known that PpIX accumulates within tumor tissue and areas without a defined BBB, such as the circumventricular organs [73]. As such, 5-ALA may selectively permeate the BBB in areas of tumoral neoangiogenesis or alterations to tumor cell transporter expression [88]. Malignant glioma is regarded as a “whole brain” disease, indicating that a clinically meaningful tumor burden is known to exist beyond what is identifiable on imaging [8,86,87,89,90]. Because of the migratory behavior of glioma cells, it is thus entirely possible that infiltrating tumor cells migrate away from the tumor center towards regions of comparatively more intact BBB, hindering 5-ALA access to the entire tumor [91]. Although the integrity of the BBB is known to be compromised in malignant glioma, the extent of disruption is the subject of debate [89]. 

In LGG, there tends to be less BBB disruption compared to HGG, which may contribute to decreased 5-ALA permeability and uptake in the former class of tumors [92,93]. Nonetheless, there is 5-ALA uptake and consequent fluorescence in a significant minority of LGG, indicating other important mechanisms of 5-ALA uptake that are tumor-dependent. A few studies have pointed that the variation can exist even within a single tumor, and seems to depend on levels of cellular proliferation [94,95,96]. Further, some studies have shown that in non-fluorescent LGG, there is still some level of resultant PpIX accumulation that is undetectable macroscopically, but detectable through spectroscopy [97,98,99]. These studies suggest that accumulation of 5-ALA past the BBB may occur through multiple mechanisms simultaneously. Which of these mechanisms predominates is not clear.

### 4.3. Tumor Microenvironmental Factors

The effects of factors unique to the tumor microenvironment have been studied in the context of 5-ALA fluorescence and PpIX accumulation. Increased temperature can stimulate increased PpIX synthesis following 5-ALA administration, this is thought to be due to the fact that the rate limiting enzyme of heme synthesis (PBGD) almost doubles in efficiency at 45 over 37 °C [66,78,100]. Hypothermia has also been employed to protect surrounding normal brain tissue during photodynamic therapy. One study demonstrated that mild hypothermia (34 °C) leads to increased PpIX accumulation, tumor fluorescence, and survival after 5-ALA administration for photodynamic therapy in a rat glioma model, contrary to prior evidence [79]. Of note, angiogenesis, a hallmark of glioma tumorigenesis, is associated with hyperthermia in the tumor [101]. pH has also been shown to influence the production of PpIX in cells exposed to 5-ALA. PpIX production is maximal at pH 7.4 and could be negatively affected by the acidic environment in which tumor cells reside [66]. Additionally, the photosensitizing effect of porphyrin derivatives in cancer cells has been shown to be oxygen dependent, an effect associated with reduced photodynamic therapy efficacy in hypoxic conditions [80,81]. Hypoxia is a hallmark of the tumor microenvironment for low- and high-grade gliomas alike. Finally, the physical microenvironment might contribute to PpIX synthesis, with one study by Niu et al. reporting higher concentrations of PpIX in glioma cells cultured on tissue-simulating gels than on stiffer tissue culture plastic [102].

## 5. Photodynamic Therapy

The principle of photodynamic therapy involves treatment with a photosensitizing compound that accumulates in a target tissue, followed by photoactivation. Photoactivation entails irradiation of the target tissue with a specified wavelength of light that catalyzes ROS production by acting on the accumulated photosensitizing compound [66]. The first step of PDT is administration of the photosensitizing compound by either venous injection or topical application depending on its intended use. After a lag period which allows for the photosensitizing agent to accumulate in the target tissues (e.g., cancer cells), light of a specific wavelength is applied to the desired area, and activation of the compound occurs, leading to local tissue destruction via ROS [103,104]. The advent of PDT dates back to early 20th century Germany and evolved to seminal experiments in the mid-20th century.

Hematoporphyrin was shown to be useful for tumor detection in 1960, and in 1978 Dougherty et al. demonstrated the clinical efficacy of hematoporphyrin-based PDT in several diverse cancers, including mycosis fungoides, angiosarcoma, and chondrosarcoma [105]. Since then, its clinical applications have expanded broadly. PDT today is most commonly known for its use in treating the dermatological condition actinic keratosis, and it has also shown efficacy in treatment of squamous cell carcinoma in situ and basal cell carcinoma, as well as severe acne and inflammatory conditions such as psoriasis, cutaneous sarcoidosis, and lichen planus [103,106,107]. In treatment of premalignant conditions and cancer, PDT has been utilized for Barrett’s esophagus, esophageal carcinoma, cervical cancer, head and neck cancer, prostate cancer (targeting vasculature), lung cancer, bladder cancer, and peritoneal cancer, among others [106,108]. Exploration of PDT as combinatorial therapy with chemotherapy, immunotherapy, and radiotherapy has shown survival benefits and quality of life improvement in treating cancer as well [109].

In malignant gliomas, PDT has been administered via stereotactic navigation to augment 5-ALA activation and associated cytotoxicity, with some retrospective evidence to suggest a benefit [110,111]. The dual effect of 5-ALA intraoperative fluorescence and photodynamic therapy has been explored as a combinatorial strategy for the treatment of glioma [110]. 5-ALA administration has allowed for improved visualization of tumors intraoperatively and improves survival in glioma patients undergoing surgical resection [45,112].

### 5.1. Cytotoxic Mechanisms

Several cytotoxic mechanisms have been described in the setting of PDT, including autophagy, apoptosis, necrosis, necroptosis, and paraptosis [113]. At lower doses, it is thought that cell survival pathways may be activated, namely autophagy. However, at higher doses of ROS, apoptosis occurs. One study describing mechanisms of apoptosis associated with 5-ALA PDT reported an increased Bax:Bcl-2 ratio and mitochondrial release of cytochrome c and apoptosis-inducing factor in U87MG GBM cells [114]. A similar study found that ALA-DT induces apoptosis via increased activity of caspase-3 and -9, increased cytochrome c, as well as decreased mitochondrial membrane potential [115]. Another study identified 5-ALA induction of receptor-interacting protein 3, a known mediator of caspase-independent apoptosis. There is also evidence to suggest that 5-ALA PDT destroys vascular endothelial cells, indirectly contributing to tumor necrosis by disrupting blood flow [116]. 5-ALA has also been reported to act as a radiosensitizer, enhancing ionizing irradiation-induced mitochondrial oxidative stress and cell death [117]. Altogether, the cell death mechanisms initiated by PDT are multifactorial and influenced by a unique mixture of programmed cell death via apoptosis as well as necrosis due to cellular injury.

### 5.2. 5-ALA PDT Immune Effects

With the exception of immunotherapies, radiotherapy and most chemotherapeutics have a net immunosuppressive effect, secondary in part to myelosuppression [118]. On the other hand, PDT is known to induce profound inflammatory effects on tumor cells. The release of heat-shock protein 70 in response to PDT is one of the most important factors in developing an immune response [118]. Specifically, these proteins form complexes with tumor antigens that are presented by antigen-presenting cells to stimulate an anti-tumor immune response [119]. In G422 murine glioma models, it has been shown that PDT increases the ratio of CD4+/CD8+ lymphocytes and promotes TNF-α and IFN-**γ** release and promotes an anti-glioma response mediated by complement C3 and T-cells [120]. Additionally, 5-ALA has been shown to accumulate in macrophages and suppress prostaglandin E_2_ production by downregulating cyclooxygenase-2 and microsomal prostaglandin E synthase-1 expression [121].

## 6. Conclusions

The use of 5-ALA for fluorescence-guided resection of malignant brain tumors has become well accepted and a routine part of clinical care in many centers. However, the molecular and metabolic mechanisms that govern the specific and differential tumor tissue fluorescence triggered by 5-ALA administration are still incompletely understood. In recent years, attempts to elucidate these mechanisms have provided new insights into the role of the heme biosynthesis pathway in both the accumulation of PpIX and in glioma biology. There are numerous steps in the 5-ALA metabolism pathway that could be targeted to modulate PpIX accumulation and, by extension, glioma tissue fluorescence. Further investigation of this pathway may facilitate deeper understanding of high-grade glioma metabolism and biology.

## Figures and Tables

**Table 1:** Potential therapeutic targets in the 5-ALA fluorescence pathway.

Potential Target	Supporting Literature	Findings
Blood–brain barrier	Novotny et al., 2000 [67]	5-ALA uptake into the choroid plexus occurs via a low pH-dependent uptake by PEPT2, and possibly a separate putative Na and HCO3-dependent mechanism
	Utsuki et al., 2006 [68]	Small, but detectable accumulations of PpIX were found in cellular boundaries of diffuse astrocytoma treated with 5-ALA using laser spectroscopy intraoperatively
	Ennis et al., 2003 [69]; Garcia et al., 1998 [70]; Malakoutikhah et al., 2010 [71]; Terr et al., 1983 [72]	The intact BBB is relatively impermeable to 5-ALA
	Olivo and Wilson, 2004 [73]	5-ALA can weakly penetrate the intact BBB leading to PpIX accumulation, but at markedly lower concentrations than areas without BBB such as circumventricular regions; PpIX accumulation is higher around tumor and inflamed brain tissue
	Molina et al., 2013 [74]	Aquaporin-4 expression was higher in fluorescent gliomas and metastatic tissues compared to non-fluorescent tissues
	Fukuda et al., 1992 [75]	Liposome encapsulated ALA administration in a mouse tumor model led to greater porphyrin accumulation compared to free ALA
Membrane transporters		
ABCB6	Zhao et al., 2013 [63]	ABCB6 expression was elevated in human glioma cell lines which correlated with an increase in intracellular PpIX accumulation
ABCG2	Kawai et al., 2019 [62]	ABCG2 expression was inversely related to 5-ALA positive staining in various non-CNS cancer cell lines; knockdown and inhibition of ABCG2 lead to increased 5-ALA cell staining
	Ogino et al., 2011 [58]	ABCG2 mediates PpIX cellular efflux and prevents PpIX in select cancer cells; inhibition of ABCG2 increased PpIX accumulation
	Oberstadt et al., 2013 [61]	ABCG2 is variably expressed in GBM
Peripheral benzodiazepine receptor (PBR)	Mesenholler et al., 2000 [59]	Competitive inhibition of the mitochondrial PBR lead to decreased PpIX accumulation in the mitochondria in a pancreatoma cell line; the PRB plays a role in PpIX translocation out of the mitochondrial membrane
	Bisland et al., 2007 [60]	Induction of PBR in glioma cell lines with low-level light treatment increased PpIX accumulation in cells
Epidermal growth factor receptor (EGFR)	Fontana et al., 2017 [64]	Co-expression of EGFR and EGFRvIII in GBM cell lines lead to activation of heme-oxygenase 1 (HO-1) and reduced cell fluorescence; inhibition of HO-1 restored fluorescence
Heme synthesis enzymes		
Ferrochelatase	Teng et al., 2011 [76]	Ferrochelatase mRNA is downregulated in glioblastoma tissue; glioma cells treated with interference RNA showed enhanced PpIX fluorescence after 5-ALA exposure
	Briel–Pump et al., 2018 [46]	Medulloblastoma cell lines treated with 5-ALA had increased PpIX enhancement associated with decreased ferrochelatase expression
	Fukuhara et al., 2013 [37]	Inhibition of ferrochelatase in human prostate cancer cell lines led to increased PpIX accumulation after 5-ALA treatment; in vivo experiments showed increased phototherapy-induced cell death after 5-ALA plus deferoxamine
	Krieg et al., 2000 [36]	Bladder carcinoma cell lines had decreased ferrochelatase and altered iron content which may be regulators of fluorescence after 5-ALA treatment
	Krieg et al., 2002 [35]	Human colon carcinoma cells showed higher fluorescence after 5-ALA treatment, higher porphobilinogen deaminase activity, and lower ferrochelatase activity compared to human fibroblasts
	Van Hillegersberg et al., 1992 [77]	Ferrochelatase activity is decreased 3-fold in a rat model of colon carcinoma liver metastases compared to normal liver cells
	Berg et al., 1996 [47]	Human adenocarcinoma and hamster fibroblast cell lines treated with iron chelators plus 5-ALA showed increased PpIX accumulation and inhibition of ferrochelatase activity
Porphobilinogen deaminase (PGDB)	Greenbaum et al., 2002 [42]	A significant fraction of PGBD localizes to glioma cell nuclei following cellular differentiation
	Greenbaum et al., 2003 [43]	In glioma cells PBGD interacts with RanBPM to induce cellular differentiation via interactions with chromatin
ALA-dehydratase (ALAD)	Hinnen et al., 1998 [39]; Navone et al., 1990 [38]	ALA-dehydratase and other heme synthesis enzyme activities are upregulated in tumor cell lines compared to normal cells
	Schauder et al., 2011 [40]	Down-regulation of either ALAD or PGDB lead to increased activity of the other enzyme and decreases in PpIX accumulation; both enzymes are important for 5-ALA induced phototherapy
Environmental factors		
Temperature	Hirschberg et al., 2004 [78]	Concurrent photodynamic therapy and hyperthermia (40–46 °C) produced a synergistic effect in inducing apoptosis in glioma spheroids
	Fisher et al., 2017 [79]	Mild hypothermia (34 °C) in a rat glioma model increased PpIX accumulation and fluorescence, increased normal neuron survival after photodynamic therapy, and extended animal lifespan
pH	Collaud et al., 2004 [66]	PpIX formation is maximized in a slightly alkaline, but physiological pH of 7.4
Oxygen	Moan and Sommer, 1985 [80]	Decreased oxygen partial pressure reduced the efficacy of photodynamic therapy in tumor cell lines
	Albert et al., 2014 [81]	5-ALA-treated glioma cell lines in atmospheric oxygen conditions (pO2 = 19%) required less irradiation to kill compared to cell lines at physiological tumor conditions (pO2 = 9%)

## Abbreviations

WHO	World Health Organization
LGG	low-grade glioma
HGG	high-grade glioma
GBM	glioblastoma multiforme
EOR	extent of resection
5-ALA	5-aminolevulinic acid
2-HG	R-2-hydroxyglutarate
α-KG	α-ketoglutarate
PpIX	protoporphyrin IX
BBB	blood–brain barrier
FECH	ferrochelatase
PBGD	porphobilinogen-deaminase
CPOX	coproporphyrinogen oxidase
PBGS	Porphobilinogen synthase
EGFR	epidermal growth factor receptor
PDT	photodynamic therapy
PEPT-2	proton-dependent peptide transporter 2
ROS	reactive oxygen species
ABCG2	ATP-binding cassette transporter G2
ABCB6	ATP-binding cassette transporter B6
TNFα	tumor necrosis factor alpha
IFN**γ**	interferon gamma
NADPH	nicotinamide adenine dinucleotide phosphate
MRI	magnetic resonance imaging
TCA	tricarboxylic acid
CNS	Central nervous system
